# Supplementary material for: Management of calcified canals during root canal treatment. A systematic review of case reports
Source: PeerJ. 2025 Sep 1;13:e19900. doi: 10.7717/peerj.19900 (PMC12422281; doi:10.7717/peerj.19900)
Supplement: Supplemental Information 3 [file peerj-13-19900-s003.pdf]

NIHR | National Institute for Health and Care Research

PROSPERO
International prospective register of systematic reviews

Management of calcified canals during root canal treatment. A Systematic Review of Case reports

KUNAL GIRI, KULVINDERSINGH BANGA, AJINKYA PAWAR

To enable PROSPERO to focus on COVID-19 submissions, this registration record has undergone basic automated checks for eligibility and is published exactly as submitted. PROSPERO has never provided peer review, and usual checking by the PROSPERO team does not endorse content. Therefore, automatically published records should be treated as any other PROSPERO registration. Further detail is provided here.

Citation
KUNAL GIRI, KULVINDERSINGH BANGA, AJINKYA PAWAR. Management of calcified canals during root canal treatment. A Systematic Review of Case reports. PROSPERO 2024 Available from https://www.crd.york.ac.uk/PROSPERO/view/CRD42023460967

REVIEW TITLE AND BASIC DETAILS

Review title
Management of calcified canals during root canal treatment. A Systematic Review of Case reports

Original language title
Management of calcified canals during root canal treatment. A Systematic Review of Case reports

Review objectives
What are the different ways of management of calcified canals during root canal treatment?

SEARCHING AND SCREENING

Searches
A systematic search following the principle of systematic review search will be carried out in the Cochrane Central Register of Controlled Trials (CENTRAL), MEDLINE, Google Scholar, PubMed using MeSH words, text words and Boolean operators. The articles in the English language will be considered. The period of publication considered will be up to 30-09-2023.

Study design

Inclusion:

In-vivo studies- case reports

Exclusion:

Clinical studies, cross-sectional studies, in-vitro and animal studies will be excluded

ELIGIBILITY CRITERIA

Condition or domain being studied

Pulp canal calcification, or sclerosis, may be the result of physiological aging of the tooth, sequela following dental trauma, surgeries (auto-transplantations), carious lesions, excessive orthodontics, iatrogenic dental treatment. The American Association of Endodontics (AAE) classifies these endodontic treatments as having a high level of difficulty, given the risk of complications or even failures. As conducting root canal treatment with calcified canals is difficult, different approaches for its management have been given in literature. The present systematic review aims to assess the different methods in management of calcified canals.

Population

Studies involving the adult population undergoing root canal treatment.

Intervention(s) or exposure(s)

Exposure: calcified root canals

Comparator(s) or control(s)

Not applicable

Context

Inclusion Criteria

- Studies published till 30/09/2023
- Studies published in English language only.
- Studies with full-text articles only will be included.
- Studies including population with calcified root canals will only be included

Exclusion Criteria

- Studies not fully available in the database.
- Cross-sectional studies, clinical trials, In-vitro and animal studies
- Studies presenting as only abstracts and not free full text articles

OUTCOMES TO BE ANALYSED

Main outcomes

Management of calcified root canals

Measures of effect

none

Additional outcomes

Not required

Measures of effect

none

DATA COLLECTION PROCESS

Data extraction (selection and coding)

Titles and/or abstracts of studies retrieved using the search strategy and those from additional sources will be screened independently by two review authors to identify studies that potentially meet the inclusion criteria outlined above. The full texts of these potentially eligible studies will be retrieved and independently assessed for eligibility by two review team members. For the assessment of each publication, spreadsheets will be used. Data will be extracted using a standardized form. Both the authors will compare each other and confirm the data.
Two review authors will extract data independently and third author will cross check the data. Discrepancies will be identified and resolved through discussion (with a third author where necessary). The data to be extracted will include: author's name, year of publication, study design, sample size, age group, Intervention description, comparison description if any, outcome measures and confounding factors if any.

Risk of bias (quality) assessment

Quality assessment of case reports will be done according to Joanna Briggs Institute (JBI) critical appraisal tool for case reports.

PLANNED DATA SYNTHESIS

Strategy for data synthesis

If similar kind of sufficient studies will be identified than Meta-analysis will be carried out.
The RevMan software will be used for the analysis.

Analysis of subgroups or subsets

Analysis of sub-groups is not planned yet.

REVIEW AFFILIATION, FUNDING AND PEER REVIEW

Review team members

- Dr KUNAL GIRI, NAIR HOSPITAL DENTAL COLLEGE
- Dr KULVINDERSINGH BANGA, NAIR HOSPITAL DENTAL COLLEGE
- Dr AJINKYA PAWAR, NAIR HOSPITAL DENTAL COLLEGE

Review affiliation

NAIR HOSPITAL DENTAL COLLEGE

Funding source

NO

Named contact

KUNAL GIRI. DEPARTMENT OF CONSERVATIVE DENTISTRY AND ENDODONTICS, NAIR HOSPITAL DENTAL COLLEGE, DR A.L. NAIR ROAD, MUMBAI CENTRAL, MUMBAI 400008

kunalgiri4@gmail.com

TIMELINE OF THE REVIEW

Review timeline
Start date: 01 November 2023. End date: 30 April 2024

Date of first submission to PROSPERO
08 September 2023

Date of registration in PROSPERO
19 September 2023

CURRENT REVIEW STAGE

Publication of review results
The intention is not to publish the review once completed.

Stage of the review at this submission

Review stage	Started	Completed
Pilot work		
Formal searching/study identification		
Screening search results against inclusion criteria		
Data extraction or receipt of IP		
Risk of bias/quality assessment		
Data synthesis		

Review status
The review is currently planned or ongoing.

ADDITIONAL INFORMATION

PROSPERO version history

- Version 1.1 published on 19 Sep 2023
- Version 1.0 published on 19 Sep 2023

Review conflict of interest
None known

Country
India

Medical Subject Headings
Environment; Humans; Root Canal Therapy; Tooth Root

Disclaimer

The content of this record displays the information provided by the review team. PROSPERO does not peer review registration records or endorse their content.

PROSPERO accepts and posts the information provided in good faith; responsibility for record content rests with the review team. The owner of this record has affirmed that the information provided is truthful and that they understand that deliberate provision of inaccurate information may be construed as scientific misconduct.

PROSPERO does not accept any liability for the content provided in this record or for its use. Readers use the information provided in this record at their own risk.

Any enquiries about the record should be referred to the named review contact
